# The Convergent Neuroscience of Affective Pain and Substance Use Disorder

**DOI:** 10.35946/arcr.v41.1.14

**Published:** 2021-12-16

**Authors:** Amanda R. Pahng, Scott Edwards

**Affiliations:** 1 Southeast Louisiana Veterans Health Care System, New Orleans, Louisiana; 2 Department of Physiology, Louisiana State University Health Sciences Center–New Orleans, New Orleans, Louisiana

**Keywords:** alcohol, cingulate cortex, insula, opioids, pain, reinforcement

## Abstract

Opioids and alcohol are widely used to relieve pain, with their analgesic efficacy stemming from rapid actions on both spinal and supraspinal nociceptive centers. As an extension of these relationships, both substances can be misused in attempts to manage negative affective symptoms stemming from chronic pain. Moreover, excessive use of opioids or alcohol facilitates the development of substance use disorder (SUD) as well as hyperalgesia, or enhanced pain sensitivity. Shared neurobiological mechanisms that promote hyperalgesia development in the context of SUD represent viable candidates for therapeutic intervention, with the ideal strategy capable of reducing both excessive substance use as well as pain symptoms simultaneously. Neurocognitive symptoms associated with SUD, ranging from poor risk management to the affective dimension of pain, are likely mediated by altered activities of key anatomical elements that modulate executive and interoceptive functions, including contributions from key frontocortical regions. To aid future discoveries, novel and translationally valid animal models of chronic pain and SUD remain under intense development and continued refinement. With these tools, future research strategies targeting severe SUD should focus on the common neurobiology between negative reinforcement and affective elements of pain, possibly by reducing excessive stress hormone and neurotransmitter activity within shared circuitry.

A central feature of substance use disorder (SUD) is the emergence of negative affective or emotional states that influence the motivational properties of misused substances.^1^ Individual propensity to experience pain-related negative affect, for example, is hypothesized to be associated with the maintenance of both opioid use disorder (OUD) and alcohol use disorder (AUD). Chronic pain is estimated to affect approximately 20% of adults worldwide,^2^ a number that will likely increase over the next several decades given the aging global population. Accordingly, opioids and/or alcohol may be sought and taken in excessive amounts to alleviate such symptoms.^3^,^4^ From a neuroanatomical perspective, ascending nociceptive circuitry is well known to interact with and alter the function of frontocortical reinforcement systems key to the development and maintenance of both OUD and AUD.^5^ The current state of neuroscience research aims not only to understand how these interactions manifest in the brain, but also to exploit these discoveries to promote novel therapeutic strategies targeting both chronic pain and SUD.^6^

This review focuses on two widely used analgesic agents, opioids and alcohol. Excessive use of either substance generates neuroadaptations that likely contribute to negative reinforcement processes in which efforts to achieve pain relief intersect with the likelihood of developing SUD, sometimes known as SUD liability.^7^ Historically, the majority of preclinical pain studies have focused on peripheral and spinal nociceptive processes, yet have produced few translational therapies for chronic pain or safe alternatives to opioid-based analgesia.^8^ Although alcohol represents another widely utilized strategy for pain relief,^9^ the many pathophysiological risks associated with heavy drinking considerably outweigh the analgesic benefits.^10^

The most recent conceptualizations and research efforts have attempted to understand the specific contributions of pain-associated negative affect to the establishment of a variety of SUD. These efforts have focused on the role of central nociceptive and motivational brain areas underlying the transition to chronic pain and its potentially crucial relationship to SUD.^11^,^12^ From a neurobiological perspective, this review describes key contributions from frontocortical areas that represent a shared neuroanatomical substrate for the intersection of pain and SUD-related symptomatology. Although this review focuses on opioids and alcohol, it is important to note that other misused substances—including nicotine and cannabis—can act as analgesics, and integrative mechanisms described in this review may play a role in the manifestation of one or more types of SUD.

## PAIN RELIEF AS NEGATIVE REINFORCEMENT IN SUD

Opioid analgesics are the most powerful and effective medications for the treatment of acute pain.^13^ Opioids are also widely accepted for use with intractable pain related to cancer or end-oflife care. Both naturally occurring (e.g., morphine) and synthetic (e.g., fentanyl) opioids produce strong and quantifiable analgesia across multiple modalities in both humans and animal models. The opioid receptors (mu, kappa, and delta) differ by the endogenous ligands that bind to them and by the range of effects the receptors produce, which is largely dependent on receptor location.^14^ The pain-relieving properties of opioids are predominately mediated by mu-opioid receptor function based on the high binding affinity of opioid analgesics to mu-opioid receptors; however, activities at both kappa- and delta-opioid receptors also mediate analgesia.^14^,^15^ Opioid analgesics also can produce euphoria and reduce negative emotional states (e.g., stress, anxiety, depression), which is attributed to the high density of opioid receptors across limbic brain regions.^16^ There is well-described evidence that acute alcohol administration also produces analgesia in both humans and animals, but to a lesser degree than opioids.^6^ From a neuropharmacological perspective, alcohol analgesia relies on the engagement of endogenous opioid signaling,^17^ but also involves additional mechanisms including G protein-activated inwardly rectifying potassium (GIRK) channel activity.^18^ A meta-analysis by Thompson and colleagues found a strong linear relationship between alcohol consumption and reported analgesia in humans.^19^ However, some limitations of the Thompson review should be noted, including its reliance on a limited number of studies (mostly in men) where effect sizes were collapsed across several pain modalities (thermal and mechanical). Moreover, no patient groups were included in the reviewed studies, highlighting the urgent need for additional work in this clinical area. Analgesia was reported to be strongest with alcohol levels that exceed the National Institute on Alcohol Abuse and Alcoholism (NIAAA) definition of binge drinking.^20^ This identifies the potential risk involved in consuming alcohol for analgesic purposes.^21^ Furthermore, authors from an empirical study examining the interaction of pain and alcohol-induced analgesia found that hazardous drinkers (determined by AUDIT-C scores) had a greater urge and intention to drink alcohol when given experimentally induced pain compared to hazardous drinkers without pain induction.^22^ This highlights an important motivational aspect of drug-induced analgesia, where acute pain can increase the desire to drink alcohol or take opioids as an active strategy for reducing pain and associated negative emotional states. For this reason, opioids and alcohol often may be used by some individuals for a combination of pain management and stress relief.

In contrast to acute pain treatment, there is limited evidence of the utility of opioid treatment for most chronic pain conditions aside from cancer pain or pain during end-of-life care.^23^ There are also serious safety concerns that need to be considered when prescribing opioids for chronic pain, including risk of developing OUD as well as acute overdose and death; for more information, see the Centers for Disease Control and Prevention’s guideline for prescribing opioids for chronic pain.^23^ With regard to alcohol, Zale and colleagues describe a curvilinear association between drinking and pain outcomes.^24^ Whereas low to moderate alcohol use is associated with analgesia, excessive drinking is associated with poorer pain trajectories over time.^24^ Low to moderate drinking was defined as drinking below government cutoffs for hazardous or excessive drinking, while excessive drinking was defined as either binge (> 4 drinks in 2 hours for women; > 5 drinks in 2 hours for men) or heavy drinking (number of drinks on any day or per week; for women, > 3 and > 7, respectively; for men, > 4 and > 14, respectively).^24^ As mentioned above, alcohol is an effective analgesic over a dose range that overlaps the NIAAA definition of “at-risk” or binge drinking limit (females, ≥ 4 drinks, and males, ≥ 5 drinks, in about 2 hours; https://www.niaaa.nih.gov/publications/brochures-and-fact-sheets/binge-drinking).^19^ If individuals limit their drinking below this point, they may achieve some analgesic efficacy with a reduced risk of later poor health outcomes. However, if they cross this line (perhaps to achieve greater analgesia), it places them at risk of eventually developing AUD and emerging hyperalgesia symptoms.

One key reason for the increasing use of opioids and alcohol for pain relief is the development of analgesic tolerance with repeated and/or extensive use. Tolerance refers to the principle that higher dose amounts of a substance are necessary to maintain the same biochemical and perceptual effects over time,^25^ which both complicates treatment regimens and heightens SUD risk. A small prospective clinical study examined the effects of short-term opioid use on analgesic tolerance and pain sensitivity in the context of chronic pain.^26^ Thermal pain thresholds and pain tolerance were assessed in individuals with chronic lower back pain, both before and after 1 month of an escalating oral morphine treatment regimen. A short-acting opioid was given prior to pain testing to examine changes to the analgesic efficacy of opioids following this 1 month of morphine treatment. Under this state, there was a significant decrease in pain thresholds and tolerance on the cold pressor test (measure of cold pain sensitivity), but no effects on heat-related pain. The rapid development of analgesic tolerance to opioids adds support to the limited clinical effectiveness of using opioids for long-term pain treatment. Tolerance to the analgesic and euphoric effects of opioids develops faster than tolerance to other physiological symptoms, including respiratory depression.^27^ This explains why the risk of respiratory depression increases with escalated opioid use or in those who formerly misused opioids heavily and renewed opioid use after a period of protracted abstinence. The development of analgesic tolerance following chronic alcohol exposure also has been well described in animal research,^6^,^17^,^28^ but there is a lack of empirical human trials investigating the impact of tolerance on alcohol’s analgesic effects.^24^ Also unknown is how analgesic tolerance promotes alcohol craving or escalation of alcohol use in attempts to maintain analgesic effects over time.

Excessive use of alcohol and/or opioids may lead to states where both analgesic tolerance and hyperalgesia symptoms coincide.^29^ Hyperalgesia is a form of pronociceptive system sensitization that behaviorally manifests as heightened pain sensitivity. Analgesic tolerance, along with the consequent escalation of analgesic use, contributes to the development of hyperalgesia, and are all hallmarks of opioid and alcohol dependence. In an opioid- and alcohol-dependent state, abstinence results in somatic withdrawal signs, pain, negative affect, and drug craving. These negative consequences can drive escalation of use over time, where negative reinforcement is the primary motivator for continued use or renewal during relapse.^1^ Carcoba and colleagues examined the role of negative affect in opioid withdrawal-induced hyperalgesia in heroin-dependent individuals.^30^ Compared to healthy controls, individuals in acute withdrawal (24 to 72 hours) and those in protracted withdrawal (~ 30 months) from heroin exhibited decreased pain thresholds and tolerance during an ischemic pain procedure. These hyperalgesic effects were heightened by viewing negative pictures (International Affective Picture System) beforehand, which elicit negative emotional states. Opioid-enhanced pain sensitivity can also play a role in cue-induced opioid craving following protracted abstinence. In another study, the cold pressor test was used to examine pain responses in abstinent individuals with a history of OUD.^31^ These individuals had shorter periods of pain tolerance and reported higher ratings of pain-related distress compared to healthy controls. There was also a positive association between pain-related distress and opioid craving. In a cross-sectional study, individuals undergoing medication-assisted treatment (MAT) with methadone or buprenorphine were examined for opioid craving and recent illicit opioid use.^32^ The investigators found that chronic pain was present in 68% of the sample and was associated with threefold higher odds of reporting craving, potentially placing this population at greater risk of relapse. Similarly, in a separate study, chronic pain levels at baseline were correlated with lower pain tolerance, greater stress reactivity during a cold pressor task, and posttest levels of opioid craving in individuals with comorbid pain and OUD.^33^ Within comorbid pain and OUD groups, individuals who currently or formerly used MAT for OUD demonstrated increases in stress-reactivity to pain compared to opioid-naïve individuals with chronic pain. Furthermore, abstinent individuals who formerly used MAT for OUD demonstrated increased stress-reactivity to pain for some measures compared to current MAT users, indicating long-lasting consequences of OUD on neurophysiological outcomes.

Similar to opioids, hyperalgesia induced by alcohol withdrawal contributes to alcohol misuse and the development of AUD.^6^ There are strong associations between alcohol consumption, pain, and pain-related disability.^34^,^35^ In a secondary analysis of two clinical trials, Witkiewitz and colleagues found that greater pain scores were associated with alcohol drinking and increases in negative affect 1 year after treatment for AUD.^36^ Using another large clinical data set, Yeung and colleagues examined the relationship between alcohol and pain interference (i.e., how pain interferes with everyday life).^35^ In this analysis, higher alcohol consumption at baseline was associated with lower pain interference at 1-year follow-up. However, the opposite was true for individuals who exhibited more AUD symptoms. For them, higher baseline alcohol consumption was significantly related to higher pain interference at 1-year follow-up, indicating that the detrimental effects of alcohol on pain interference may emerge as the severity of the disease progresses. There is also a strong association between alcohol consumption, chronic pain, and pain-associated disability. Among persons with chronic pain, disabling pain was strongly associated with their level of alcohol consumption.^37^ There is some evidence that chronic pain status may be predictive of future drug and alcohol use. In prospective epidemiological studies, self-reported pain interference was predictive of AUD development,^38^ and persistent pain was associated with increased odds of opioid use (adjusted odds ratio [*AOR*] = 5.4) and heavy alcohol use (*AOR* = 2.2) compared to no pain.^39^

With human research, it is very difficult to determine the direction of causality for the relationship between SUD and pain. Fortunately, a major benefit of animal research is the care with which experimental conditions can be controlled to determine the direction of causality for these complex associations. Preclinical animal research has been critical for the modeling of interactions between pain and SUD, and some of the most widely used techniques are described here.

## ANIMAL MODELS TO EXAMINE PAIN AND SUD INTERACTIONS

Key symptomatology of OUD and AUD—including escalation of drug intake, compulsive drug seeking, development of hyperalgesia, and the emergence of negative affective states—can be reliably modeled in rodents. When discussing drug-induced hyperalgesia, it is necessary to discriminate that nociception and pain are different phenomena. Nociception refers to the neural process of encoding noxious stimuli, whereas pain refers to a personal experience that is influenced by biological, psychological, and social factors. Pain is therefore a subjective and inherently emotional experience. Accordingly, the empirical assessment of pain in rodents can be challenging. It is possible, however, to assess nociception and affective pain-like behavior in rodents through a variety of assays. Preclinical animal models also provide valuable tools for investigating the somatic and behavioral symptoms of SUD, identifying neurobiological changes associated with SUD, and testing medications to alleviate symptoms of dependence and reduce abuse liability. These models impact medication development and increase understanding of the behaviors that contribute to the development of SUD. There are several different procedures for inducing opioid and alcohol dependence in animals. Most involve the general procedure of repeatedly putting animals through a period of intoxication where the drug is administered by the experimenter or self-administered by the animals. This is followed by a period where the drug is not available, which produces a state of spontaneous withdrawal. As this cycle of intoxication and withdrawal is repeated, animals will begin to exhibit symptoms of dependence, including escalation of intake (if the drug is self-administered), pain-like behavior, compulsive drug-seeking behavior, and the emergence of negative emotional states (e.g., anxiety-like behavior).^40^ When the drug is administered by the experimenter, the behavioral and neurochemical consequences of drug escalation can be mimicked by giving animals an escalating dose regimen to achieve a state of dependence.^40^ In rodents, the most commonly used routes of administration for opioids include intravenous self-administration and subcutaneous administration, while the routes of administration for alcohol include oral self-administration, ethanol vapor exposure, intragastric gavage, a liquid diet containing alcohol (e.g., Lieber-DeCarli diet), and intraperitoneal administration.

### Measurement of Nociception and Affective Pain in Animals

There are numerous tests to assay pain-like behavior in rodent models of psychiatric disease,^41^ although the most common tests of nociceptive behavior in the context of hyperalgesia include von Frey^42^ and Hargreaves^43^ tests of mechanical hypersensitivity and thermal hypersensitivity, respectively. These reflexive-based tests involve applying a mechanical or thermal stimulus to the rodent’s hind paw and measuring either the paw withdrawal threshold (typically in grams of pressure) for a graded mechanical stimulus or the paw withdrawal latency (typically in seconds) for a constant thermal stimulus. A higher paw withdrawal threshold or latency compared to baseline is associated with an analgesic or anti-nociceptive process (e.g., following administration of an opioid substance), while a lower paw withdrawal threshold or latency is associated with hyperalgesia (i.e., more sensitive to the stimulus when compared to baseline). As discussed earlier, the subjective pain experience can greatly impact motivational processes associated with the transition to SUD. One shortcoming of these reflexive-based assays is the inability to assess the motivational and affective dimensions of pain, which are hypothesized to influence the transition to both chronic pain states^44^ and SUD.^45^,^46^ Neuroscientists are beginning to employ additional behavioral tests that attempt to more closely assess the cognitive and motivational aspects of pain-like behavior beyond the somatic or sensory components. These non-reflexive-based assays allow the potential to examine the contribution of negative affective-like states towards activity avoidance and pain interference in the context of SUD.^47^,^48^ In the mechanical conflict-avoidance system (MCS) task, animals traverse mechanically noxious probes of varying heights to avoid a bright aversive light, escaping to reach a goal chamber that is dark. A longer latency to exit onto the probes reflects increased pain avoidance-like behavior as a motivational correlate of hyperalgesia. The specific strengths and limitations of the MCS procedure have been described, illustrating its utility in measuring both analgesic and hyperalgesic conditions.^47^,^49^,^50^ Another innovative technique in this area is the Orofacial Pain Assessment Device (OPAD), which pairs a thermal stimulus conflict with access to an appetitive reward^51^ and can be readily applied to oral alcohol or opioid self-administration. These reflex-based and non-reflex-based pain assays can be used in tandem to more comprehensively examine the effects of opioid and alcohol dependence on both somatic and affective pain-like behaviors in rodents.

### Measurement of Opioid-Induced Hyperalgesia in Animals

Induction of opioid dependence in rodents can be achieved through intravenous self-administration where animals are given extended (or long) access (LgA; 6 hr, 12 hr, or 24 hr) versus limited (or short) access (ShA, 1 hr) to opioids,^52^ including prescription opioids such as fentanyl and oxycodone.^53^ In this model, LgA animals exhibit hallmarks of OUD including escalation of opioid intake, compulsive opioid seeking, development of hyperalgesia, and the emergence of negative emotional states. Male Wistar rats given LgA (12 hr) to heroin self-administration (0.06 mg/kg/infusion) exhibit decreased paw withdrawal thresholds compared to ShA (1 hr) animals during spontaneous withdrawal, indicating opioid-induced mechanical hyperalgesia.^54^ Interestingly, the emergence of opioid-induced hyperalgesia coincided with escalated heroin intake in LgA animals, which was not observed in ShA animals.^54^ In this study, increased heroin intake was significantly correlated with increased pain-like behavior (lower paw withdrawal thresholds), demonstrating the close relationship between opioid intake and pain symptoms in the context of dependence. Repeated subcutaneous administration of opioids can also induce dependence and pain-like behavior in rodents. Rats given repeated subcutaneous doses of heroin for 5 days exhibited decreased paw withdrawal thresholds compared to animals given a single dose of heroin, demonstrating the ability of opioids to drive nociceptive system sensitization.^29^ In a separate study, male Wistar rats were given an escalating dose regimen of morphine (10 mg/kg to 20 mg/kg) over 2 weeks to examine the effects of morphine dependence on the sensory and motivational/affective components of pain-like behavior, using von Frey and MCS procedures, respectively.^49^ Opioid-dependent animals exhibited an increased latency to exit onto a bed of noxious mechanical probes during withdrawal compared to saline-injected controls, indicating increased pain-like avoidance with escalated morphine use. There was a modest but significant correlation between changes in mechanical hypersensitivity and pain-like avoidance behavior, indicating that the von Frey and MCS procedures examine overlapping, but not identical, measures of pain-like behavior. Continued investigations that shed light on individual differences in opioid and pain sensitivity along both somatic and affective dimensions also may help researchers to maximize the beneficial use of opioid analgesics while minimizing OUD liability.

### Measurement of Alcohol-Induced Hyperalgesia in Animals

The somatic and affective symptoms of AUD can be reliably modeled in rodents using chronic intermittent ethanol vapor (CIEV) exposure.^55^ The intermittent procedure involves daily cycles of alcohol vapor (producing peak blood alcohol levels of 150–200 mg/dl) and alcohol withdrawal. After several weeks of CIEV, alcohol-dependent male Wistar rats exhibited decreases in paw withdrawal thresholds during spontaneous withdrawal compared to non-dependent controls, indicating alcohol-induced mechanical hyperalgesia.^54^ In a separate study, 4 weeks of CIEV produced thermal hyperalgesia in alcohol-dependent male Wistar rats compared to nondependent controls.^56^ This increase in pain-like behavior was attenuated following either alcohol administration by the experimenter or alcohol self-administration. The anti-hyperalgesic effects of acute alcohol treatment in alcohol dependence provides strong evidence of the motivation to drink alcohol to ameliorate withdrawal symptoms and decrease pain. In a nonforced contingent ethanol vapor self-administration study, male Wistar rats were allowed to nose poke for ethanol vapor (8 hr/day) over either 8 or 24 sessions, which produced nonescalated and escalated nose poking for ethanol vapor exposure, respectively.^57^ Like the previous CIEV studies, rodents who escalated nose pokes demonstrated decreased paw withdrawal thresholds during withdrawal compared to nonescalated animals, indicating increased pain-like behavior. Additional models of alcohol dependence, including chronic intermittent two-bottle choice and the Lieber–DeCarli diet, produced mechanical and thermal hyperalgesia in male Sprague Dawley rats,^58^,^59^ and the “Drinking in the Dark” procedure facilitated hyperalgesia in female and male C57BL/6J mice.^60^

### Examining How Pain Influences Opioid and Alcohol Use in Animals

Another interesting area of preclinical pain research involves examining the effects of persistent pain on drug abuse liability. Neuropathic pain, fibromyalgia, low back pain, and osteoarthritis are common medical conditions that contribute to the burden of chronic pain disorders. Accordingly, preclinical models of neuropathic pain (e.g., spared nerve injury, spinal nerve ligation) and inflammatory pain (e.g., complete Freund’s adjuvant [CFA]) are frequently used to examine the effects of chronic pain on behavior and neurochemistry in rodents. Martin and colleagues found that, compared to controls, nerve-injured male Fisher 344 rats required higher amounts of heroin to maintain heroin self-administration and were more sensitive to mu-opioid receptor antagonist-induced increases in heroin self-administration.^61^ In a study examining how persistent inflammatory pain alters morphine preference, CFA reduced the number of morphine conditioning sessions required to acquire morphine-conditioned place preference in male Wistar rats.^62^ Hiplito and colleagues found that CFA altered heroin self-administration in a dose-dependent manner in male Sprague Dawley rats.^63^ High unit doses (0.2 mg/kg/infusion) were more reinforcing, and low unit doses (0.05 mg/kg/infusion) were less reinforcing. These preclinical examinations provide evidence for the hypothesis that the driving force for motivation to self-administer opioids in individuals with an underlying pain condition may be in part to seek relief from chronic pain. These findings may also indicate that shared neural substrates promote both substance use and pain chronification, or the process by which acute pain becomes chronic, as discussed in the next section.

A number of additional studies have examined the effects of chronic pain on alcohol consumption in rodents.^64^ Sciatic nerve–injured CD1 male mice consumed more alcohol (20% ethanol) and exhibited increased anxiety-like behavior compared to sham-operated mice, suggesting that a chronic pain state drives increased alcohol consumption.^65^ In a mouse model of osteoarthritis, male C57BL/6J mice consumed significantly more alcohol than sham controls during a two-bottle choice test of escalating alcohol concentrations (2.5% to 20%).^66^ During a 20% ethanol continuous access test, CFA increased alcohol drinking in male C57BL/6J mice, but did not increase drinking in female C57BL/6J mice.^67^ In contrast to these findings in mice, a recent study found no effect of CFA on alcohol self-consumption or alcohol preference in male Wistar rats.^68^ However, this study discovered that the relationship between alcohol drinking levels and hyperalgesia symptoms reversed between acute (1-week) and chronic (3-week) periods post-CFA administration, suggesting that either the motivational or analgesic effects of alcohol may be altered over the time course of chronic pain.

Altogether, there appear to be clear effects of chronic pain on opioid intake, motivation for opioids, alcohol consumption, and alcohol preference that are largely dependent on factors including rodent species and sex. In summary, repeated and extensive exposure to opioids and alcohol promotes escalation of intake and pain-like behavior, which are sequelae that can in turn exacerbate abuse liability and SUD disease severity.

## SHARED FRONTOCORTICAL SUBSTRATES FOR AFFECTIVE PAIN AND SUD

In addition to somatosensory elements, both affective/emotional and cognitive/motivational dimensions can augment pain-related morbidity.^6^ Chronic pain can generate continual negative affective states and promote new cognitive strategies and behaviors to avoid pain. Consequently, pain relief itself activates reward circuitry and is experienced as a positively valenced emotional state.^69^ It is thus hypothesized that the emergence of painful states following chronic or excessive opioid or alcohol exposure facilitates negative reinforcement processes whereby individuals seek relief from pain by escalating use of these substances, culminating in the development of psychiatric sequelae including SUD.^45^,^46^ Specific alterations in frontocortical activity may facilitate pain and promote maladaptive behaviors in close association with pain-related negative affective states. As such activity is heavily impacted by chronic or excessive opioid and alcohol exposure, further interrogation of within- and between-circuit neuroadaptations is warranted to better understand the pathological intersection of pain and SUD.^46^,^70^

## INSULAR AND CINGULATE CORTICES AND AFFECTIVE PAIN PROCESSING

The insular cortex and the cingulate cortex represent key components of a distinct neural network within the larger executive control system of the prefrontal cortex. Communication within these areas is hypothesized to facilitate attribution of emotional salience to both internal and external stimuli, including pain-related noxious stimuli.^9^ Of particular interest is the role of frontocortical regions in higher nociceptive processing, as well as their historical association with SUD.^5^ Pain is a multidimensional experience, which comprises both sensory and affective-motivational components.^71^ Through studies of these regions both in isolation and as a functional network, the insula and cingulate have been identified as key areas for supraspinal processing of the affective dimension.^18^ Imaging studies have also identified heightened activity in the insula and cingulate with the anticipation of pain and have correlated perceived pain intensity with degree of concurrent activity in the insula and cingulate in human subjects.^72^,^73^ In rodent models, selective lesions of the cingulate have been shown to reduce pain-related aversion without altering the sensory element of noxious stimuli.^74^,^75^ The insula has reciprocal connections with the cingulate and receives nociceptive information directly from the thalamus.^76^ Moreover, insula connectivity with subcortical regions such as the amygdala may facilitate emotional arousal to noxious stimuli.^76^,^77^

Resting-state functional magnetic resonance imaging (fMRI) analyses have identified a precise network based in the insula and cingulate that extends to several subcortical regions referred to as the salience network. The salience network model was developed from the integration of multiple human fMRI studies that ultimately led to the hypothesis that this particular circuitry recognizes and assimilates interoceptive and external information, recruits and derecruits additional executive networks to engage the appropriate cognitive processes (focusing attention to stimuli, including noxious stimuli), and ultimately regulates an adaptive behavioral response.^78^ Alterations in the salience network are observed in individuals with chronic pain and are associated specifically with greater pain catastrophizing,^79^ a phenomenon that is believed to be closely related to the chronification of pain. The network has most commonly been investigated in human and nonhuman primate models, but was recently confirmed in rodents, validating crucial contributions from the insula and cingulate cortex.^80^

## DYSREGULATION OF THE SALIENCE NETWORK BY ALCOHOL AND OPIOIDS

Research has provided evidence that AUD dysregulates activity of the insula-cingulate salience network in humans, typically indicated by fMRI analyses. This alteration is believed to impair executive function, compromising the ability to make appropriate or cognitively demanding decisions.^81^ Salience network deficits may specifically contribute to the maintenance or exacerbation of AUD by making an individual unable to clearly discern risky behaviors, such as the decision to seek out and consume excessive amounts of alcohol despite adverse consequences. This network may be particularly vulnerable in AUD patients exposed to stressful conditions due to cingulate dysfunction.^82^ Investigators have also found that excessive drinking may disrupt normal associations between interoception and pain.^83^ A similar involvement of endogenous opioid signaling in salience network function is well known.^84^ Alterations in the network’s connectivity are related to resting state dysfunction^85^ as well as to relapse behaviors^86^ in patients with OUD. More studies are needed to examine salience network activity in populations with OUD in relation to hyperalgesia symptoms, especially because pain symptoms can promote opioid craving even after months of abstinence.^31^

Although the salience network is most commonly examined in humans, several preclinical animal studies have begun to examine the importance of this construct with relation to pain and alcohol exposure. Interestingly, in mice, the insula and cingulate were discovered to have a role in the social transfer of pain associated with hyperalgesia following alcohol withdrawal.^87^ Another recent study found several interbrain regional correlations of glucocorticoid receptor (GR) phosphorylation in animals experiencing a binge alcohol withdrawal episode in the context of chronic inflammatory pain.^68^ The insular cortex acted as a hub for these correlations with other nociceptive regions investigated (including the cingulate cortex and central amygdala), suggesting coordinated activity in insula circuitry and glucocorticoid signaling in the context of pain and alcohol withdrawal. This type of within-subject molecular analysis at the animal level may model human fMRI analyses of related network activity. These circuit-based relationships also have been hypothesized to play a key role in the motivational processes relevant to SUD.^5^ Finally, a recent conceptual review postulated that neurovisceral feedback and interoceptive dysregulation by opioids and alcohol can be traced to alterations in gut microbiota,^88^ highlighting the need for further investigation of the gut-brain axis in SUD and related pain.

## BRAIN STRESS SIGNALING IN AFFECTIVE PAIN AND SUD

Given that chronic and unmitigated pain represents a significant stressor, elucidation of chronic opioid-and alcohol-induced neuroadaptations within brain stress systems may provide valuable insights into potential mechanisms underlying the transition to SUD in vulnerable individuals. Indeed, the role of central stress hormone and neuropeptide signaling in response to stress has emerged as a conceptual bridge between chronic substance use, affective and cognitive disruption, and propensity to relapse.^89^ As the key integrative link between the systemic and central brain stress response, the hypothalamic-pituitary-adrenal (HPA) axis is responsible for orchestrating adaptive processes that return an organism to homeostasis following exposure to a stressor. Release of corticotropin-releasing factor (CRF) from the hypothalamus initiates this process by regulating the production and processing of pro-opiomelanocortin from the anterior pituitary. The pro-opiomelanocortin transcript produces two key peptides related to the effective management of both stress (adrenocorticotropic hormone) and pain (beta-endorphin), illustrating the close relationships between these two vital physiological systems. Adrenocorticotropic hormone acts to facilitate the production and release of glucocorticoids from the adrenal cortex, after which the systemic response is under the control of critical negative and positive feedback mechanisms, whereby glucocorticoids can inhibit or stimulate (respectively) their own genomic and nongenomic actions by binding to GRs in the brain.^90^ Stress sensitization via potentiated GR signaling may represent one mechanism for intensification of SUD-associated negative affective symptoms, termed hyperkatifeia.^46^

Alcohol-dependent animals display a functional increase in brain GR signaling that appears to emerge during the transition to dependence.^91^ GR antagonism reduces escalated drinking in both preclinical animal models and in individuals suffering from AUD.^92^ It is also interesting that systemic administration of the GR antagonist mifepristone alleviates mechanical hyperalgesia symptoms observed in animals fed an alcohol diet.^93^ These convergent findings suggest that targeting excessive stress signaling may be capable of treating both excessive drinking and pain symptoms in the context of AUD. Less is understood about these associations in relation to OUD, although similar relationships connecting negative reinforcement processes to pain and OUD have been proposed.^94^,^95^ These conceptualizations are supported by research indicating links between serum cortisol levels and opioid withdrawal in humans^96^ and functional activation of negative reinforcement brain centers in opioid-dependent animals.^97^ Although systemic CRF_1_ receptor antagonism has been shown to alleviate hyperalgesia symptoms in opioid-dependent animals,^54^ no studies have investigated the potential contribution of GR signaling in this process. Given the role of chronic stress and glucocorticoid activity in exacerbating pain,^98^ additional work is necessary to determine the relationships between stress hormone signaling and pain symptoms in patients suffering from AUD and OUD.

## CONCLUSIONS

Few effective therapies exist for SUD or chronic pain. The accretive pathophysiology and shared neurobiological interactions of these disease states likely complicate their effective treatment. Powerful reinforcement processes maintain the use of opioids and alcohol to manage pain as well as the negative affective states that underlie chronic pain experiences. Future translational research priorities should aim to bridge gaps in our understanding of how opioids and alcohol act on nociceptive and higher motivational circuitry to drive tolerance and hyperalgesia symptoms that may exacerbate SUD. Numerous symptoms are regularly associated with severe SUD, ranging from poor risk management to the cognitive/affective dimension of pain. These symptoms are likely driven by neuroadaptations within key anatomical elements that regulate higher executive functions, including key contributions from the cingulate and insula cortices.
